# An inventory of the foliar, soil, and dung arthropod communities in pastures of the southeastern United States

**DOI:** 10.1002/ece3.7941

**Published:** 2021-07-22

**Authors:** Ryan B. Schmid, Kelton D. Welch, Jonathan G. Lundgren

**Affiliations:** ^1^ Ecdysis Foundation Estelline SD USA

**Keywords:** bioinventory, functional guild, grasslands, insect community, rangelands

## Abstract

Grassland systems constitute a significant portion of the land area in the United States and as a result harbors significant arthropod biodiversity. During this time of biodiversity loss around the world, bioinventories of ecologically important habitats serve as important indicators for the effectiveness of conservation efforts. We conducted a bioinventory of the foliar, soil, and dung arthropod communities in 10 cattle pastures located in the southeastern United States during the 2018 grazing season. In sum, 126,251 arthropod specimens were collected. From the foliar community, 13 arthropod orders were observed, with the greatest species richness found in Hymenoptera, Diptera, and Hemiptera. The soil‐dwelling arthropod community contained 18 orders. The three orders comprising the highest species richness were Coleoptera, Diptera, and Hymenoptera. Lastly, 12 arthropod orders were collected from cattle dung, with the greatest species richness found in Coleoptera, Diptera, and Hymenoptera. Herbivores were the most abundant functional guild found in the foliar community, and predators were most abundant in the soil and dung communities. Arthropod pests constituted a small portion of the pasture arthropod communities, with 1.01%, 0.34%, and 0.46% pests found in the foliar, soil, and dung communities, respectively. While bioinventories demand considerable time, energy, and resources to accomplish, the information from these inventories has many uses for conservation efforts, land management recommendations, and the direction of climate change science.

## Graphical abstract

We conducted a bioinventory of the foliar, soil, and dung arthropod communities in 10 cattle pastures located in the southeastern United States. In sum, 126,251 specimens were collected, representing 20 orders. Herbivores were the most abundant functional guild found in the foliar community, while predators were most abundant in the soil and dung communities.

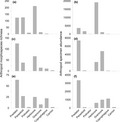

## 1 INTRODUCTION

Biodiversity declines are a significant consequence of the Anthropocene (Dirzo et al., 2014; Johnson et al., 2017; Wagner, 2020). Major drivers of change in biological community composition during the Anthropocene include habitat loss (Marini et al., 2012), climate change (Andrew et al., 2013), and agriculture intensification (Tscharntke et al., 2012). Arthropod communities are not immune to these effects, experiencing the largest population fluctuations in recorded history, with steep declines of many functional groups (Hallmann et al., 2017; Leather, 2018; Lister & Garcia, 2018; Sánchez‐Bayo & Wyckhuys, 2019; Wagner, 2020). As drivers of biological community change do not appear likely to abate in the near future, vigilant monitoring of arthropod communities is essential for facilitating conservation efforts and stemming biodiversity declines. Inventories of biological communities (bioinventories) provide vital records for future assessments of biodiversity fluctuations as the Anthropocene continues.

Rangeland/pasture systems constitute >413 million acres in the United States, comprising ≈22% of the contiguous United States (NASS, 2017). Owing to the large footprint of pastures on the terrestrial landscape, management decisions made in pasture systems have important implications for arthropod diversity (Jerrentrup et al., 2014; Wallis De Vries et al., 2007). Historically, pastures once hosted robust arthropod communities (Rottman & Capinera, 1983; Walkden & Wilbur, 1944), providing refuge from intensively managed cropland in regions of the United States (Schmid et al., 2015). The diversity of flora in pastures provides a strong resource base and a wide variety of ecological niches for arthropod communities, making these grazed grasslands important reservoirs of arthropod biodiversity, in addition to their uses in supporting agricultural production (Dennis et al., 1998; Morris, 2000; Wallis De Vries et al., 2007). The vegetation alone provides a diversity of microclimates, pollen, seeds, nectaries, and vegetation that attract a myriad of arthropods (Lundgren, 2009). Arthropods inhabiting grassland ecosystems fulfill needed ecological functions to maintain ecosystem stability and productivity for livestock production (Joern & Laws, 2013; Whiles & Charlton, 2006). In the southeastern United States, bioinventories of invertebrates in pasture systems have been relegated to surveys of economically important arthropod groups (e.g., dung beetles, red imported fire ants, ticks, and pest flies), while being confined to small regions or states (Fiene et al., 2011; Kaufman & Wood, 2012; Kramer et al., 1985; Pompo et al., 2016; Steele, 2016; Wilson, 1963). Consequently, arthropod assemblages in pastures of the Southeast region of the United States remain poorly described. This inventory serves livestock producers in the region and also serves as important biodiversity data during a pivotal time of arthropod decline. In this study, the arthropod community and functional guilds of foliar, soil, and dung arthropod communities found in Southeastern U.S. pastures are described.

## 2 METHODS

### 2.1 Study sites

**FIGURE 1:**
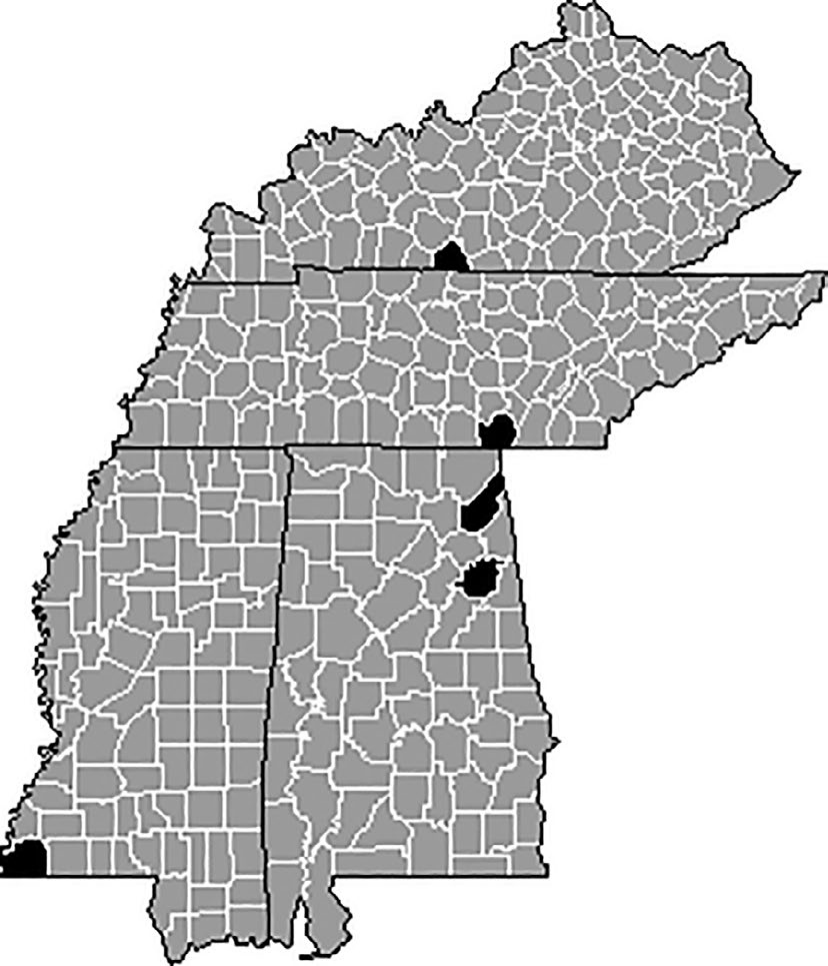
Pastures (*n* = 10) sampled for this study were located in Allen County, KY; Marion County, TN; DeKalb County, AL; Calhoun County, AL; and Wilkinson County, MS. Counties where sampling occurred are highlighted in black on state county maps

Arthropod communities were sampled from pastures (*n* = 10) located in four states of the southeastern United States: Kentucky (*n* = 2), Tennessee (*n* = 2), Alabama (*n* = 4), and Mississippi (*n* = 2) (Figure 1). Sampling the foliar and soil arthropod communities occurred three times during the 2018 grazing season (1–4 May, 23–28 July, and 29 September–October 3), while dung community samples were collected only during the July and September sampling dates. Grazing systems had been practiced on pastures for at least 10 years prior to this study, but specific cattle management practices varied among pastures (Table 1).

**TABLE 1:** Pasture usage history and cattle management practices from 2018 for adaptive multipaddock (AMP) and conventional grazed (CG) pastures in the southeastern United States

Site #	Location (county, state)	Land use history prior to current grazing management	Current grazing management	Length of current management (years)	Insecticide inputs	Herbicide inputs	Average stocking density (AU/ha)	Average paddock size (ha)	Average grazing period goals (days)	Paddock rest versus grazed period ratio
1	Allen County, KY	Tobacco & grain crops before grazing >30 years	AMP	13	Spot apply cattle with Ivermec as needed (≈1 in 80 head/year)	None	62.35	1.84	2	44.00
2	Allen County, KY	Tobacco & grain crops	CG	6	Wormer applied to cattle (product unknown) & organophosphate/pyrethroid ear tags	None	0.79	14.00	365	0.00
3	Marion County, TN	Row cropped, hay, and grazing	AMP	12	None	None	115.57	0.98	2	44.00
4	Marion County, TN	Row cropped, hay, and grazing	CG	>25	Ivermec applied to cattle & organophosphate/pyrethroid ear tags	1.25 gal/acre (1×/year) 2, 4D	1.79	15.25	135	1.67
5	DeKalb County, AL	Small grains	AMP	29	Spot apply cattle with cydectin as needed once in Dec.	None	93.00	1.67	1	59.00
6	DeKalb County, AL	Small grains	CG	17	Permethrin insecticide applied to cattle once a year	None	1.76	28.33	365	1.14
7	Calhoun County, AL	Cotton	AMP	24	No wormers or insecticide applied to cows, cydectin applied to calves	None	465.41	0.30	1	122.00
8	Calhoun County, AL	Cotton	CG	>40	Ivermec applied to cattle	None	1.94	18.00	365	1.00
9	Wilkinson County, MS	Tobacco & grain crops before grazing >50 years	AMP	10	Cydectin, pyrethroids, & organophosphates applied to cattle	None	156.25	1.44	1	149.00
10	Wilkinson County, MS	Tobacco, cotton, market gardening, & grain crops	CG	38	Ivermec applied to cattle	None	7.65	9.29	75	6.00

### 2.2 Sampling procedure

Two sampling areas (1,390 m^2^ per sampling area) were established 100 m apart, on average, in each pasture, with each sampling area containing three transect lines (45.7 m), for a total of six transects per pasture. The transects within a sampling area were parallel to one another and spaced 15.2 m apart.

Foliar‐dwelling arthropods were sampled from pasture foliage at the 22.9‐m mark of each of the three transect lines from the first sampling area in each pasture (*n* = 3 sweep samples/pasture). Vegetation was swept with a 38‐cm‐diameter net, with 25 sweeps occurring perpendicular to each side of a transect line (total of 50 sweeps per transect). All arthropods collected from individual sweep samples were stored in plastic bags containing 3 ml of 70% isopropyl alcohol to preserve and prevent specimens from cannibalism. Samples were kept on ice in the field and returned to the laboratory, where they were stored at −18℃ until arthropod specimens could be separated from loose vegetation in the sample. After which, specimens were preserved in 70% isopropyl alcohol for curation.

Core sampling (10 cm diameter, 10 cm deep) was used to collect the soil and dung communities. Cores were extracted at 7.6 and 38.1 m on two of the transect lines for soil arthropod community sampling (*n* = 8 soil cores/ranch). Dung arthropod community cores were taken from the center of randomly selected dung pats found within the pasture borders (*n* = 5 dung cores/ranch). Age of dung pats ranged from 2 to 5 days old, as this age of pat has peak arthropod abundance and diversity (Pecenka & Lundgren, 2018). All cores were kept cool on ice upon extraction from the field until they could be returned to the laboratory (60 hr). Once in the laboratory, soil and dung cores were subjected to a Berlese funnel extraction system for 7 days, which ensured each soil/dung core had completely dried and all arthropods had evacuated from the core. Arthropods extracted from cores with the Berlese system were stored in 70% isopropyl alcohol, until they could be identified and cataloged.

### 2.3 Community composition

To characterize the arthropod communities, each specimen from the foliar, soil, and dung samples was identified to the lowest taxonomic level possible. Identification of specimens was made using a variety of taxonomic keys from the literature and consultation of taxonomic experts as needed. Due to time constraints, no effort was made to identify mites (Arachnida: Acari) beyond the class level, Protura beyond the class level, thrips (Insecta: Thysanoptera) beyond the ordinal level, Symphyla beyond the class level, millipedes (Diplopoda: Julida) beyond the ordinal level, Diplura beyond the family level, nor springtails (Hexapoda: Collembola) beyond the family level. All other specimens were separated to genus or species level. Those for which positive species identifications could not be provided were assigned to a numbered morphospecies. Larvae of holometabolous insects were assigned morphotaxa independent from adult species, and treated as different morphotaxa in all analyses, owing to their discrete differences in ecological function. However, immature hemimetabolous insects were excluded from the data because they are frequently not ecologically distinct from adults and could not be reliably associated with particular adult species. Morphospecies were assigned to functional guilds, based on knowledge and current hypotheses regarding the ecology of these organisms. We recognized nine nonexclusive guilds: predator, parasitoid, pollinator, herbivore, granivore, coprophage, carrion, livestock pest, and other/unknown.

Voucher specimens are deposited in the Mark F. Longfellow Ecological Collection and housed at Blue Dasher Farm (Estelline, South Dakota, USA).

## 3 RESULTS

### 3.1 Foliar arthropod community

**FIGURE 2:**
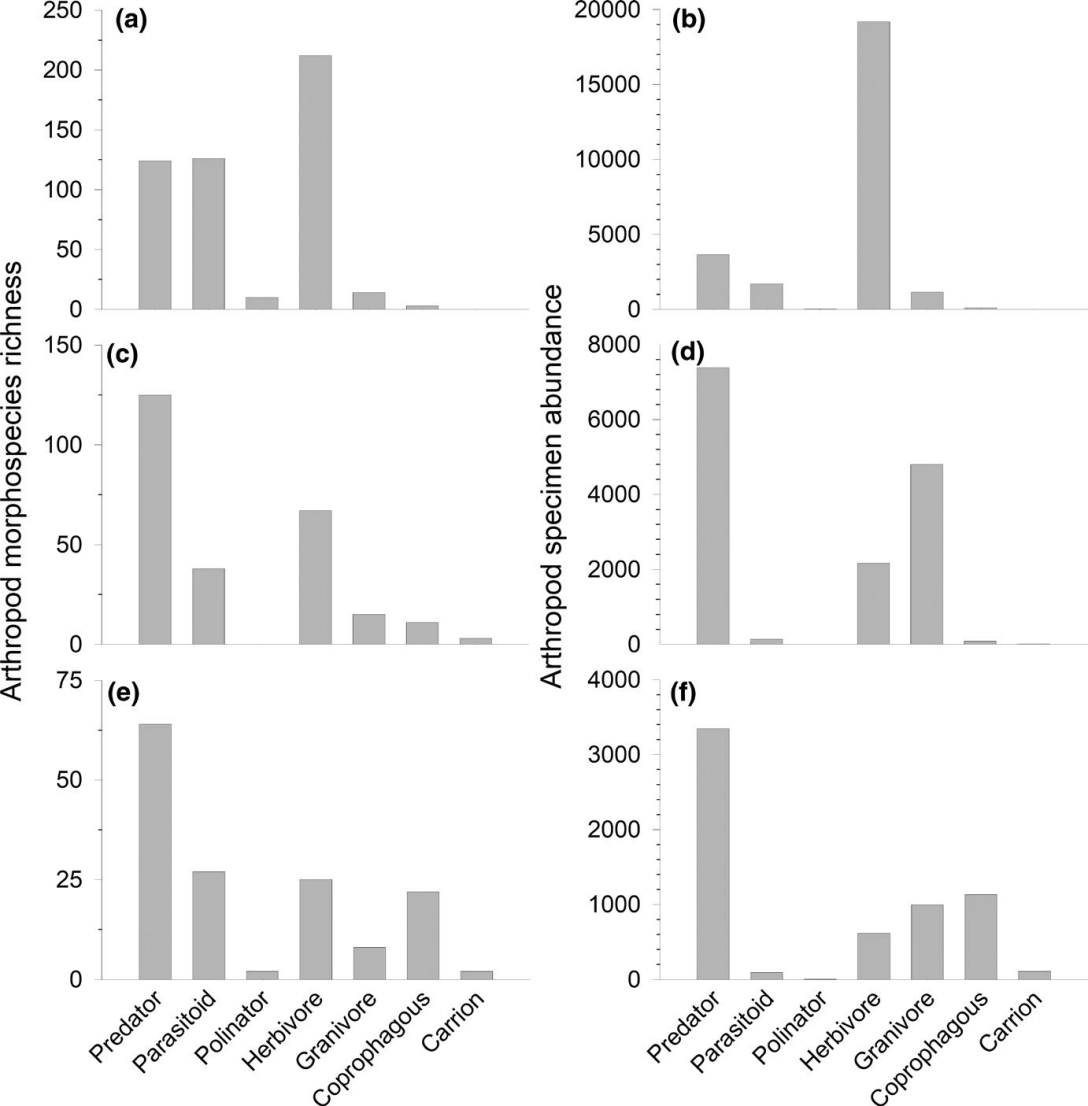
Functional guilds of arthropod species richness and specimen abundance for foliar (a, b), soil (c, d), and dung (e, f) communities. Arthropod communities were sampled from pastures (*n* = 10) grazed by cattle in the southeastern United States from May to October 2018. Due to the abundance of specimens with unknown functional guild status, owing to a lack of documentation in the scientific literature, specimens with unknown functional guilds were excluded from the figure

**FIGURE 3:**
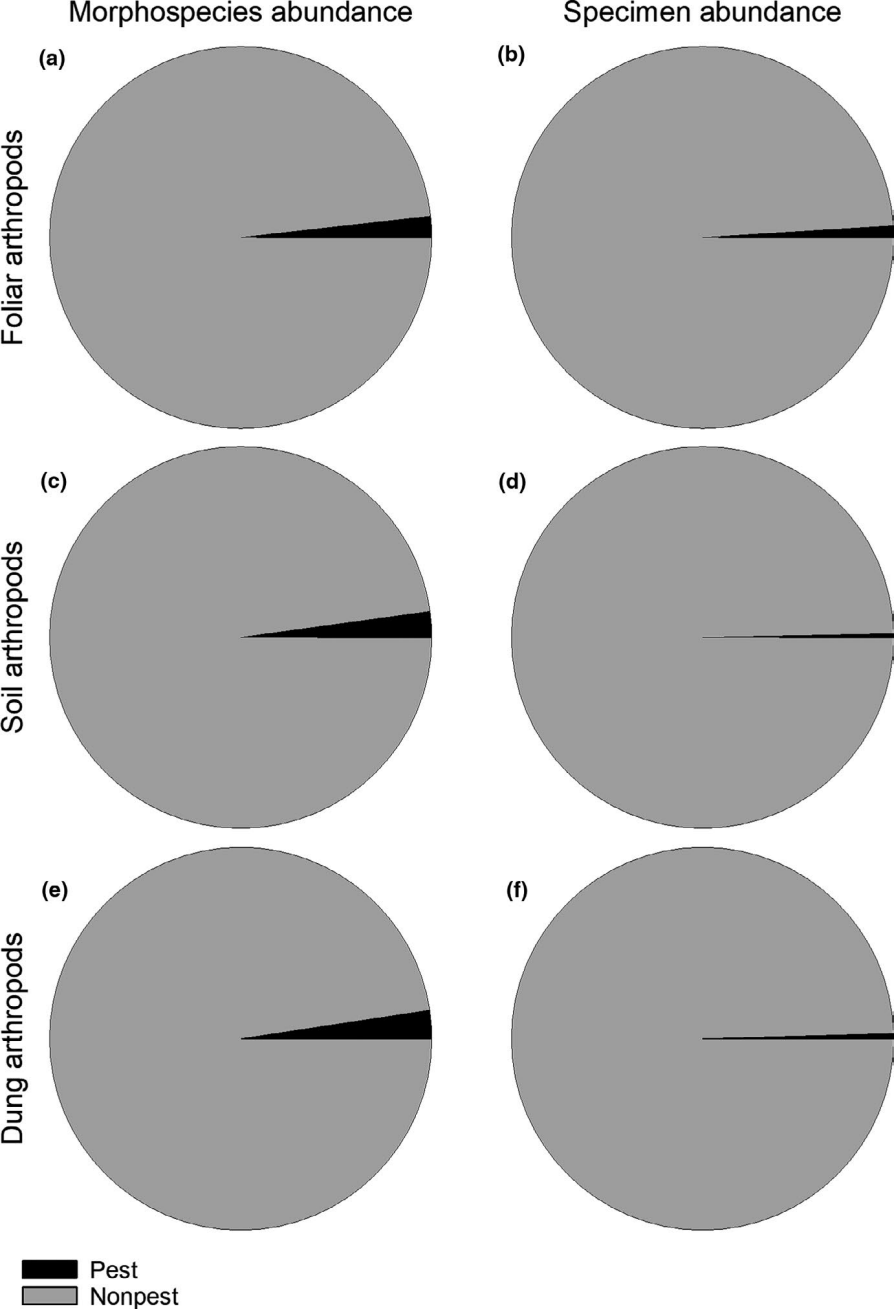
Abundance of pest species in terms of morphospecies and specimens collected from foliar, soil, and dung arthropod communities. Arthropods were collected from cattle‐grazed pastures (*n* = 10) in the southeastern United States

In total, 52,128 arthropod individuals were collected from pasture foliage, representing 759 morphospecies from four classes (Arachnida, Collembola, Insecta, and Symphyla) and 13 orders (Araneae, Coleoptera, Diptera, Entomobryomorpha, Hemiptera, Hymenoptera, Lepidoptera, Neuroptera, Odonata, Orthoptera, Psocoptera, Symphypleona, and Thysanoptera). A complete list of foliar arthropod specimens and their abundance from this study can be found in the Supporting Information accompanying this article. The five orders that contained the highest number of morphospecies and specimen abundance are listed in Table 2. Ecological guilds of the foliar arthropod community consisted predominately of herbivores, parasitoids, and predators (Figure 2a,b). Only 1.84% of the morphospecies and 1.01% of the specimen abundance were pests (Figure 3a,b).

**TABLE 2:** The top 5 arthropod orders for species richness (morphospecies) and abundance of specimens collected (abundance) in foliar, soil, and dung habitats

	Morphospecies			Abundance	
Foliar	1)	Hymenoptera	194	Hemiptera	17,601
	2)	Diptera	163	Diptera	11,440
	3)	Hemiptera	155	Hymenoptera	3,386
	4)	Coleoptera	105	Orthoptera	2,831
	5)	Araneae (spiders)	55	Araneae (spiders)	1,924
Soil	1)	Coleoptera	172	Hymenoptera	7,105
	2)	Diptera	100	Hemiptera	2,500
	3)	Hymenoptera	54	Coleoptera	1,503
	4)	Hemiptera	53	Diptera	1,075
	5)	Araneae (spiders)	23	Araneae (spiders)	201
Dung	1)	Coleoptera	102	Coleoptera	3,215
	2)	Diptera	53	Hymenoptera	1,456
	3)	Hymenoptera	37	Diptera	1,342
	4)	Hemiptera	13	Hemiptera	544
	5)	Araneae (spiders)	5	Araneae (spiders)	31

Note

Arthropods were collected from cattle‐grazed pastures (*n* = 10) in the southeastern United States from May to October 2018.

### 3.2 Soil arthropod community

A total of 224 soil cores were extracted for this study to examine the soil‐dwelling arthropod community. In sum, 53,292 arthropod individuals were extracted from the soil, representing 436 morphospecies from eight classes (Arachnida, Chilopoda, Collembola, Diplopoda, Diplura, Insecta, Protura, and Symphyla) and 18 orders (Araneae, Coleoptera, Dicellurata, Diptera, Dermaptera, Entomobryomorpha, Geophilomorpha, Hemiptera, Hymenoptera, Julida, Lepidoptera, Lithobiomorpha, Neuroptera, Opiliones, Orthoptera, Psocoptera, Symphypleona, and Thysanoptera). A complete list of soil arthropod specimens and their abundance from this study can be found in the Supporting Information accompanying this article. The five orders containing the highest number of morphospecies and specimen abundance are listed in Table 2. Examining the known functional guilds of arthropod morphospecies shows predators comprised the largest portion of the community followed by herbivores and parasitoids (Figure 2c). Predators also comprised the largest functional guild of known arthropod abundance followed by granivores and herbivores (Figure 2d). Pest species constituted only 2.25% of the morphospecies and 0.34% of the specimen abundance (Figure 3c,d).

### 3.3 Dung arthropod community

A total of 100 dung pats were subjected to core sampling. In sum, 20,831 arthropod individuals, representing 234 morphospecies from six classes (Arachnida, Chilopoda, Collembola, Diplura, Insecta, and Symphyla) and 12 orders (Araneae, Coleoptera, Dermaptera, Dicellurata, Diptera, Entomobryomorpha, Hemiptera, Hymenoptera, Orthoptera, Psocoptera, Symphypleona, and Thysanoptera). A complete list of dung arthropod specimens and their abundance from this study can be found in the Supporting Information accompanying this article. The five orders with the highest number of morphospecies and specimen abundance are listed in Table 2. Predators were the most speciose functional group, followed by parasitoids and herbivores (Figure 2e). Predators were also the most abundant of the functional guilds, followed by coprophages and granivores (Figure 2f). Only 2.47% of the morphospecies and 0.46% of the specimen abundance were pests (Figure 3e,f).

## 4 DISCUSSION

Our survey shows an abundant and diverse arthropod community in pastures of the southeastern United States. In total, 126,25 specimens were identified to morphospecies for this survey, making it one of the largest assessments of arthropod diversity in pasture systems in the region. Previous surveys of arthropods in grazing lands of the southeastern United States focused on specific groups of arthropods (e.g., pests, dung beetles, biocontrol agents, and pollinators) or on specific habitats within pastures (e.g., plant foliage or dung pats) (Hu, 1995; Kramer et al., 1985; Leppla et al., 2017; Pompo et al., 2016; Wilson, 1963). To our knowledge, our bioinventory is one of the most comprehensive surveys of arthropod communities in pastures of the southeastern United States, including foliage, soil, and dung microhabitats. Furthermore, this bioinventory identified the majority of collected arthropods to the family level. The magnitude and comprehensive identification of specimens from our bioinventory will serve as a valuable reference for future biodiversity studies.

Our bioinventory shows both similarities and differences in community composition compared to previous studies conducted in the region. For example, a survey of dung‐dwelling arthropods in north‐central Florida found the orders with the highest species richness to be Coleoptera (109 species), Diptera (35 species), and Hymenoptera (24 species) (Hu, 1995), while our study found a similar pattern of species richness in the dung community with Coleoptera (105 species), Diptera (53 species), and Hymenoptera (40 species). The higher number of Diptera and Hymenoptera species reported by our study relative to Hu (1995) may be the result of the larger geographic range conducted by our survey. Another example of arthropod bioinventories in grasslands of the Texas panhandle found the canopy‐dwelling to have the highest specimen abundance in the orders of (a) Hemiptera, (b) Araneae, (c) Orthoptera, (d) Coleoptera, and (e) Hymenoptera (Bhandari et al., 2018). These results differ from our survey, which documented the five most abundant orders to be (a) Hemiptera, (b) Diptera, (c) Hymenoptera, (d) Orthoptera, and (e) Araneae. While Hemiptera tops the list of both bioinventories, the remainder of the orders differ between the two inventories. The differences in arthropod abundance between the inventories could be for a multitude of reasons, for example, method of sampling, time of sampling, grassland management, and surrounding landscape. One likely mechanism that contributed to the differences between inventories was variances in grassland habitat between the two distant geographic locations, that is, the Southern High Plains versus the southeastern United States. The results of previous arthropod bioinventories relative to our bioinventory highlight the differences that exist in arthropod communities from similar habitats but different geographic locations. This information underpins the need for future arthropod inventories from various habitats and different regions as a means to accurately assess biodiversity declines during the Anthropocene.

The assemblage of functional guilds was distinct among the three arthropod communities sampled for this study (Figure 2). To begin, herbivores were the most abundant guild in the foliar community, while predators were the most abundant guild in the soil and dung communities. The high abundance of herbivores in the foliar community is not surprising, as herbivores are often the most abundant group found in plant canopies of grasslands (Cagnolo et al., 2002; Hironaka & Koike, 2013). However, the relatively low abundance of pollinators in the foliar community was unexpected to us. But this result does align with a recent rangeland bioinventory, which documented pollinators to be only a minor portion of the community (Bhandari et al., 2018). The abundance of pollinators in pasture systems is likely influenced by forb abundance. Consequently, the small number of pollinators may be a result of pasture management that prioritizes production of grasses over forbs for cattle production. Although grass production is the primary concern of ranchers raising cattle, a balanced cattle diet includes forbs (Grant et al., 1985). Thus, increasing the abundance of forbs in pastures could improve pollinator abundance, while also fulfilling cattle dietary requirements. Unlike the foliar community, the soil and dung communities were dominated by predatory arthropods. Due to the lack of experimental manipulation in the design of this study, it is difficult to ascertain why predators were the most abundant group in the soil and dung. However, it should be noted that a significant portion of the collected specimens were classified as unknown in terms of functional guild. We hypothesize that many of the unknown arthropods in our survey constituted food sources for the predator population, helping to support the large predator portion of the community. We hypothesize many of the unknown specimens are detritivores, but lack creditable sources to classify these specimens. It is also worth noting that pest species comprised a very small portion of the arthropod community in all three habitats, comprising only 1.01%, 0.34%, and 0.46% of arthropod abundance in the foliar, soil, and dung habitats, respectively. The predators combined with competition from specimens of other functional guilds seem to be capable of holding pest populations in check in these pasture systems. This phenomenon has been observed in pasture systems in the southeastern United States before, with predators and parasitoids significantly increasing horn fly mortality (Hu, 1995). Further studies are needed to better understand life histories of many of the organisms and further our understanding of arthropod food webs in pasture ecosystems.

Describing arthropod communities of various habitats and agriculture systems requires further attention in order to provide meaningful data for future studies (Apfelbaum & Haney, 2012; Goldstein, 2004). For instance, bioinventories of pastures can inform land managers and government agencies about livestock and land management practices that promote conservation efforts of endangered species or guilds of conservation interest, for example, rusty‐patch bumblebee or pollinators. Furthermore, bioinventories serve as reference points to compare shifts in community composition as novel pasture management methods are tested. Perhaps most importantly, bioinventories serve as a reference during biodiversity declines of the Anthropocene. Without bioinventories, we are essentially flying blind during this time of biodiversity loss. Leaving us unable to make informed decisions to conserve at‐risk species. While bioinventories demand considerable time, energy, and resources to accomplish, they are vital for the future of biodiversity on our planet, and their value cannot be overstated.

## CONFLICT OF INTEREST

The authors declare no conflict of interest.

## AUTHOR CONTRIBUTIONS

**Ryan B. Schmid:** Conceptualization (equal); Data curation (lead); Writing‐original draft (lead); Writing‐review & editing (lead). **Kelton D. Welch:** Formal analysis (supporting); Project administration (supporting); Writing‐original draft (supporting); Writing‐review & editing (supporting). **Jonathan G. Lundgren:** Conceptualization (equal); Funding acquisition (lead); Writing‐original draft (supporting); Writing‐review & editing (supporting).

## Supporting information

Supplementary Material

## Data Availability

Inventory of collected arthropods from foliar, soil, and dung is available on Dryad (https://doi.org/10.5061/dryad.ns1rn8ptc).
